# Investigation of the diagonal elements of the Wigner’s reaction matrix for networks with violated time reversal invariance

**DOI:** 10.1038/s41598-019-42123-y

**Published:** 2019-04-04

**Authors:** Michał Ławniczak, Leszek Sirko

**Affiliations:** Institute of Physics, Polish Academy of Sciences, Aleja Lotników 32/46, 02-668, Warsaw, Poland

## Abstract

The distributions of the diagonal elements of the Wigner’s reaction $\hat{K}$ matrix for open systems with violated time reversal *T* invariance in the case of large absorption are for the first time experimentally studied. The Wigner’s reaction matrix links the properties of chaotic systems with the scattering processes in the asymptotic region. Microwave networks consisting of microwave circulators were used in the experiment to simulate quantum graphs with violated *T* invariance. The distributions of the diagonal elements of the reaction $\hat{K}$ matrix were experimentally evaluated by measuring of the two-port scattering matrix $\hat{S}$. The violation of *T* invariance in the networks with large absorption was demonstrated by calculating the enhancement factor *W* of the matrix $\hat{S}$. Our experimental results are in very good agreement with the analytic ones attained for the Gaussian unitary ensemble in the random matrix theory. The obtained results suggest that the distributions *P*(*ʋ*) and *P*(*u*) of the imaginary and the real parts of the diagonal elements of the Wigner’s reaction $\hat{K}$ matrix together with the enhancement factor *W* can be used as a powerful tool for identification of systems with violated *T* symmetry and quantification of their absorption.

## Introduction

Quantum chaotic scattering was originally introduced in order to describe processes of nuclear scattering^1^. It is of great interest for understanding properties of large scale complicated quantum systems^2–4^, however, their controllable experimental investigation is difficult and sometimes impossible. For that reason multitude of complicated physical problems from the field of quantum chaos are best tackled experimentally with the help of microwave networks (graphs) simulating quantum graphs^5,6^. To emphasize the nomenclature equivalence between microwave networks and microwave graphs we will use both names interchangeably. Quantum graphs as structures of vertices connected by edges were first studied by Linus Pauling^7^. Their usefulness stems from the fact that they can be considered as practical models of real physical networks. Quantum graphs provide extremely rich platform for studying properties of bounded quantum systems which are chaotic in the classical limit^5,8–15^ and open systems which display chaotic scattering^6,16–18^.

To the large variety of systems and models described by quantum graphs belong, e.g., superconducting quantum circuits^19^, quantum circuits in tunnel junctions^20^, experimental setups to realize high-dimensional multipartite quantum states^21^, discrete-time quantum gravity models^22^ and functional connectivity in preclinical Alzheimer’s disease^23^.

The microwave networks (graphs) simulate quantum graphs^8,9,13^ because there is a direct analogy between the telegraph equation describing a microwave network and the Schrödinger equation of the corresponding quantum graph^5,24^. This is the only system which allows for the experimental simulation of quantum systems corresponding to all three classical ensembles in the random-matrix theory (RMT): with *T* invariance belonging to Gaussian orthogonal ensemble (GOE)^5,17,25,26^ and Gaussian symplectic ensemble (GSE)^27^ as well as systems without *T* invariance belonging to Gaussian unitary ensemble (GUE)^5,6,28–30^.

Properties of open chaotic systems with *T* invariance (symmetry index *β* = 1 in RMT) were comprehensively investigated in many important aspects. The statistical distributions of a single port scattering matrix *S* in the models including imperfect coupling and direct processes were investigated theoretically and experimentally in refs^31–37^.

The distributions *P*(*ʋ*) and *P*(*u*) of the imaginary and the real parts of the Wigner’s reaction matrix as well as the reflection coefficient *P*(*R*) were theoretically investigated for all ranges of the dimensionless parameter *γ* = 2*π*Γ/Δ, characterizing the absorption strength^38,39^, where Γ and Δ are the width of resonances and the mean level spacing, respectively. For microwave chaotic cavities they were experimentally studied in refs^40–42^ and for microwave graphs, in the case of medium and large absorption strength *γ* ≤ 47.7, they were investigated in refs^17,43–45^. It is important to point out that the distribution of the imaginary *P*(*ʋ*) parts of the diagonal elements of the Wigner’s reaction matrix is known in solid-state physics as the local density of states (LDoS)^38^. The enhancement factor *W* was also studied using microwave networks^6^, where the investigations were focused on the absorption strength *γ* < 54.4. One should point out that quantum graphs with leads, which are other interesting open objects, were in details theoretically studied in refs^12,16,18,46^.

A different situation exists for open chaotic systems with violated time reversal invariance (symmetry index *β* = 2 in RMT) and large absorption. Such systems have been only fragmentarily experimentally studied so far. In refs^6,47^ the enhancement factor was investigated for microwave networks without *T* invariance for the absorption strength 7 < *γ* < 62.

Therefore, in this paper we discuss the first experiment which deals with the important characteristics of open chaotic systems - the distributions of the diagonal elements of the Wigner’s reaction matrix $\hat{K}$^38,48^. The Wigner’s reaction $\hat{K}$ matrix links the properties of chaotic systems (reaction regions) with the scattering processes in the asymptotic region. One should mention that off-diagonal entries to the Wigner reaction matrix $\hat{K}$ were theoretically studied for T-invariant systems in the limiting case of zero absorption in ref^49^ while the full predictions for the distribution of the off-diagonal entries of the $\hat{S}$ matrix was given in ref^50^. The distributions of the diagonal elements of the 2 × 2 reaction $\hat{K}$ matrix can be obtained from the normalized two-port scattering matrix $\hat{s}$ of the investigated system which is evaluated in the case of perfect coupling, when direct processes are not present^36,42^

1 $$\hat{K}=i\frac{\hat{s}-\hat{I}}{\hat{s}+\hat{I}},$$

where $\hat{I}$ is the 2 × 2 identity matrix. The relationship between the matrix $\hat{s}$ and the two-port scattering matrix $\hat{S}$ measured directly in the experiment will be discussed in details later. The matrix $\hat{K}$ is also related to the normalized impedance^42^ $\hat{z}:\,\hat{K}=-\,i\hat{z}$.

Our studies are focused on microwave networks without *T* invariance in the limit of large absorption. One should point out that the two-port measurements are also indispensable because at large absorption the convectional measures of *T* violation such as short- and long-range spectral correlation functions^51^, e.g., the nearest neighbor level spacing distribution or the level variance are useless because the individual levels are not resolved. In such a case the enhancement factor *W* can be used as a sensitive measure of *T* invariance violation^6^. The evaluation of the enhancement factor *W* requires the measurements of the full two-port scattering matrix

2 $$\hat{S}=[\begin{array}{cc}{S}_{11} & {S}_{12}\\ {S}_{21} & {S}_{22}\end{array}].$$

The diagonal elements of $\hat{S}$ can be parameterized as

3 $${S}_{ii}=\sqrt{{R}_{i}}{e}^{i{\theta }_{i}},$$

where *θ*_*i*_ and *R*_*i*_ are the phase and the reflection coefficient measured at the *i*^*th*^ port of the network.

In the experimental investigations quantum graphs were modeled by microwave networks (graphs). The analogy between microwave graphs and quantum graphs stems from the equivalency of the telegraph equation which describes the microwave circuits and the Schrödinger equation describing the quantum systems with the same topology^5^.

A microwave graph contains vertices (microwave joints) connected by edges, e.g., coaxial cables. In the present investigations the SMA-RG402 coaxial cables were applied. The SMA-RG402 coaxial cable has a center conductor of radius *r*_1_ = 0.05 cm encompassed by a tubular Teflon insulating layer having a dielectric constant $\varepsilon \simeq 2.06$^52,53^. The insulating layer is encompassed by a tubular conductor of radius *r*_2_ = 0.15 cm. One should point out that inside a coaxial cable below the outset of the TE_11_ mode can propagate only the fundamental transverse-electromagnetic (TEM) mode. For the SMA-RG402 coaxial cable the TE_11_ mode cut-off frequency is ${\nu }_{cut}\simeq \frac{c}{\pi ({r}_{1}+{r}_{2})\sqrt{\varepsilon }}\simeq 33$ GHz^54^, where *c* is the speed of light in vacuum. Absorption of the networks was effectively controlled by adding to the networks microwave attenuators 1 dB and 2 dB, respectively^43,44^.

**Figure 1:**
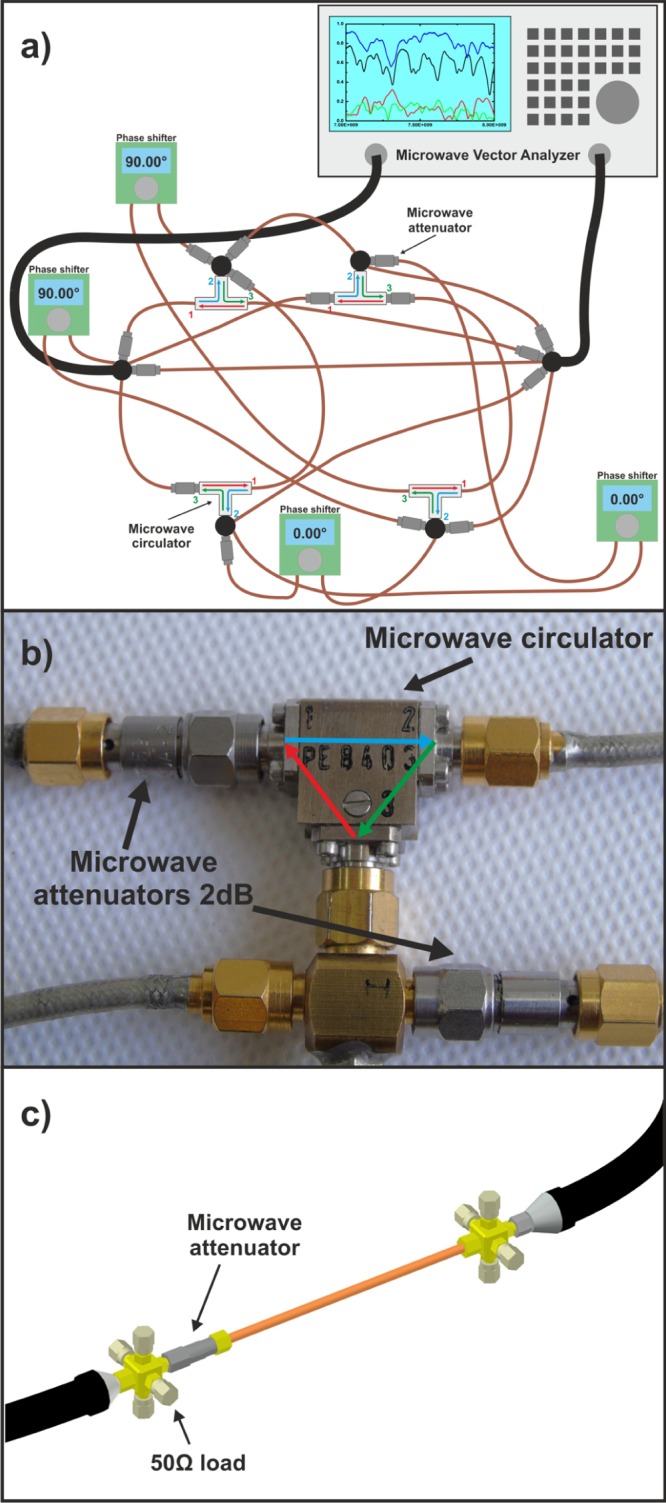
The experimental setups for the evaluation of the Wigner’s $\hat{K}$ matrix. (**a**) The schematic diagram of the experimental setup for measuring the scattering matrix $\hat{S}$ of the fully connected microwave networks (graphs) with violated *T* invariance and absorption. (**b**) The *T* violation was induced with four Anritsu PE8403 microwave circulators. 1 dB and 2 dB attenuators were used to vary absorption in the graphs. Here we show 2 dB attenuators. (**c**) The schematic of the setup for measuring of the two-port radiation scattering matrix ${\hat{S}}^{r}$. The matrix ${\hat{S}}^{r}$ was measured at the inputs of the 6-joint vertices. In order to simulate removed to infinity vertices 50 Ω loads were connected to the four connectors of these joints. The fifth connector of the 6-joint vertex was connected to fifth connector of the another 6-joint vertex in order to account for the direct processes between the 6-joints.

The two-port scattering matrix $\hat{S}$ of fully connected hexagon graphs required for evaluation of the Wigner’s matrix $\hat{K}$ and the enhancement factor *W* was measured using the setup shown in Fig. 1(a). The *T* violation was induced with four Anritsu PE8403 microwave circulators with low insertion loss which operate in the frequency range from 7–14 GHz. These are non-reciprocal three-port passive devices. A wave entering the circulator through port 1, 2 or 3 exits at port 2, 3, or 1, respectively, as illustrated schematically in Fig. 1(b). Ensembles of different microwave networks realizations were created by changing the lengths of four edges of the networks using the phase shifters visible in Fig. 1(a).

A vector network analyzer (VNA), Agilent E8364B, was used to measure the scattering matrix $\hat{S}$ of the hexagon microwave graphs in the frequency range 7–14 GHz. The networks were connected to the VNA through the leads - HP 85133-616 and HP 85133-617 flexible microwave cables - connected to 6-joint vertices. The other four vertices of the graphs were 5-joints. Figure 1(a) shows also that to increase absorption of the networks each edge of the network contained a microwave attenuator.

In order to identify *T* symmetry of the investigated system we used the elastic enhancement factor *W*^6,36,55–62^ of the two-port scattering matrix $\hat{S}$ which is defined by the following relation

4 $$W=\frac{\sqrt{{\rm{var}}({S}_{11}){\rm{var}}({S}_{22})}}{{\rm{var}}({S}_{12})},$$

where *var*(*S*_12_) ≡ 〈|*S*_12_|^2^〉 − |〈*S*_12_〉|^2^ stands for the variance of the matrix element *S*_12_. It was established that the enhancement factor *W* for $\gamma \gg 1$ is not influenced by the direct processes present in the system^55,63–65^. For large absorption the enhancement factor *W* depends weakly on the parameter *γ*^36,55^, approaching for $\gamma \gg 1$ the limit of *W* = 2/*β*.

In this study we will consider the system in the regime of large absorption where the effective parameter *γ*, which includes the contributions from large internal absorption and two open channels, will be evaluated using the single channel distribution *P*(*R*) of the reflection coefficient *R*. It is possible because in the large absorption limit the distribution *P*(*R*) can be well approximated by the exponential Rayleigh distribution^36^ which no longer depends explicitly on the number of open channels^40,66^. This property of the distribution *P*(*R*) will be discussed further in more detail. Moreover, we will show that the same role may play the distributions of the imaginary and the real parts of the diagonal elements of the $\hat{K}$ matrix.

For systems without *T* invariance (*β* = 2), the analytic expression for the distribution of the reflection coefficient *R* is given by^35,39,65^

5 $$P(R)=\frac{2}{{\mathrm{(1}-R)}^{2}}{P}_{0}(\frac{1+R}{1-R})\mathrm{.}$$

The probability distribution *P*_0_(*x*) is defined by

6 $${P}_{0}(x)=\frac{1}{2}[A{(\frac{\alpha (x+1)}{2})}^{\beta /2}+B]\,\exp (-\frac{\alpha (x+1)}{2}),$$

where *α* = *γβ*/2, *A* = *e*^*α*^ − 1 and *B* = 1 + *α* − *e*^*α*^.

In the large absorption limit the formula (5) can be well approximated by the exponential Rayleigh distribution^36^

7 $$P(R)=\alpha {e}^{-\alpha R}.$$

The probability distribution *P*_0_(*x*) can be also used for calculating the distributions of the imaginary and the real parts *P*(*ʋ*) and *P*(*u*) of the diagonal elements of the Wigner’s $\hat{K}$ matrix^38^

8 $$P(\upsilon )=\frac{\sqrt{2}}{\pi {v}^{3/2}}{\int }_{0}^{\infty }\,dq{P}_{0}[{q}^{2}+\frac{1}{2}(\upsilon +\frac{1}{v})],$$

and

9 $$P(u)=\frac{1}{2\pi \sqrt{{u}^{2}+1}}{\int }_{0}^{\infty }dq{P}_{0}[\frac{\sqrt{{u}^{2}+1}}{2}(q+\frac{1}{q})],$$

where −*ʋ* = *ImK*_*ii*_ < 0 is the imaginary and *u* = *ReK*_*ii*_ is the real part of the *i*^*th*^ diagonal element of the Wigner’s reaction matrix.

In the large absorption limit the formulas (8) and (9) are read as follows^36^

10 $$P(\upsilon )=\sqrt{\frac{\alpha }{4\pi {v}^{3}}}\,\exp \,[-\frac{\alpha }{4}{(\sqrt{\upsilon }-\frac{1}{\sqrt{\upsilon }})}^{2}],$$

and

11 $$P(u)=\sqrt{\frac{\alpha }{4\pi }}\,\exp \,[-\frac{\alpha {u}^{2}}{4}].$$

## Results

For each realization of a microwave graph the absorption strength $\gamma =\frac{1}{2}{\sum }_{i=1}^{2}\,{\gamma }_{i}$ was evaluated by fitting the theoretical mean reflection coefficient

12 $${\langle R\rangle }^{th}={\int }_{0}^{1}\,dRRP(R),$$

to the experimental one $\langle {R}_{i}\rangle =\langle {s}_{ii}{s}_{ii}^{\dagger }\rangle$ obtained after eliminating the direct processes^36,37,43^. Here the index *i* = 1, 2 denotes the port 1 or 2. Since we deal with microwave systems the direct processes can be also eliminated using the impedance approach^41,42^. In this very elegant method the normalized two-port network scattering matrix $\hat{s}$, with no direct processes present (perfect coupling case), can be calculated using the formula

13 $$\hat{s}=(\hat{I}-\hat{z})/(\hat{I}+\hat{z}),$$

where $\hat{I}$ is the 2 × 2 identity matrix and the normalized impedance $\hat{z}$ of a chaotic microwave network is given by

14 $$\hat{z}={({\rm{Re}}{\hat{Z}}^{r})}^{-1/2}[{\rm{Re}}\,\hat{Z}+i({\rm{Im}}\,\hat{Z}-{\rm{Im}}\,{\hat{Z}}^{r})]{({\rm{Re}}{\hat{Z}}^{r})}^{-1/2}.$$

The formulas for the network and the radiation impedance matrices are following: $\hat{Z}={\hat{Z}}_{0}^{1/2}(\hat{I}+\hat{S})/(\hat{I}-\hat{S}){\hat{Z}}_{0}^{1/2}$ and ${\hat{Z}}^{r}={\hat{Z}}_{0}^{1/2}(\hat{I}+{\hat{S}}^{r})/(\hat{I}-{\hat{S}}^{r}){\hat{Z}}_{0}^{1/2}$. They are expressed by the network $\hat{S}$ and the radiation ${\hat{S}}^{r}$ scattering matrices, respectively. ${\hat{z}}_{0}$ is a real 2 × 2 diagonal matrix of characteristic impedance of the network edges attached to the 6-joint vertices. The two-port radiation scattering matrix ${\hat{S}}^{r}$ was measured at the inputs of the 6-joint vertices. In this case 50 Ω loads were connected to the four connectors of these joints to simulate the vertices removed to infinity. The fifth connector of the 6-joint vertex was connected to fifth connector of the another 6-joint vertex in order to account for the direct processes between the 6-joints. The schematic diagram of the setup for evaluating the radiation matrix ${\hat{S}}^{r}$ of the 6-joint vertices is shown in Fig. 1(c). Moreover, what is the most important in this analysis, the diagonal elements of the Wigner’s reaction matrix $\hat{K}$ can be evaluated using the formula (1).

**Figure 2:**
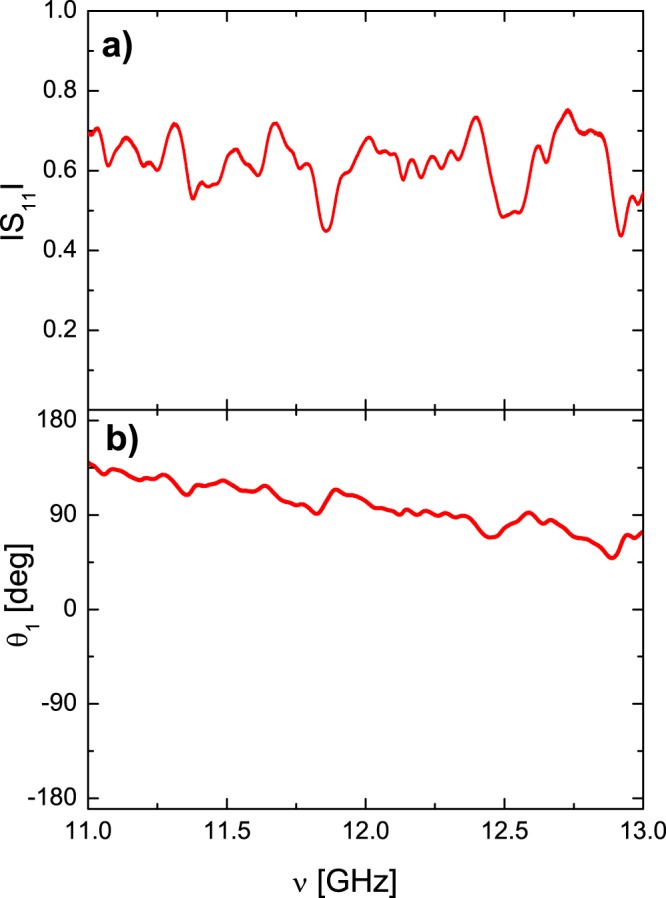
An example of the measured *S*_11_ element of the two-port scattering matrix $\hat{S}$. Panels (a) and (b) show the modulus |*S*_11_| and the phase *θ*_1_ of the scattering matrix $\hat{S}$ measured for the microwave graphs with violated *T* invariance and *γ* = 48.4 in the frequency range 11–13 GHz. The measurements were done for the graphs containing 2 dB attenuators. The total “optical” length of the graph including edges, joints, phase shifters, microwave attenuators and circulators was 789 cm.

Figure 2 shows the examples of the modulus |*S*_11_| and the phase *θ*_1_ of the diagonal element *S*_11_ of the matrix $\hat{S}$ of the microwave graph with *γ* = 48.4. The measurements were done for the network containing 2 dB attenuators in the frequency range 11–13 GHz. Its total “optical” length including phase shifters, circulators, joints and attenuators was 789 cm.

**Figure 3:**
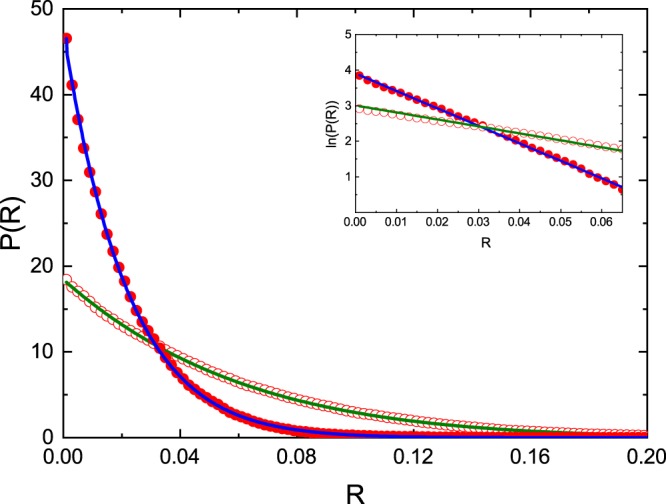
Experimentally evaluated distributions of the reflection coefficient *P*(*R*) for the microwave graphs with violated *T* invariance at *γ* = 19.4 (red open cirles) and *γ* = 48.4 (red full circles). They are compared with the theoretical ones calculated from the Eq. (5). The theoretical results are denoted by green (*γ* = 19.4) and blue (*γ* = 48.4) solid lines, respectively. The inset shows the distributions of the reflection coefficient *P*(*R*) in semi-log scale. The straight lines *ln*(*P*(*R*)) = *aR* + *b* fitted to the experimental results yielded the slopes *a* = −19.6 ± 0.2 and *a* = −48.9 ± 0.5 for the networks with 1 dB and 2 dB attenuators, respectively.

In Fig. 3 the experimental distributions of the reflection coefficient *P*(*R*) for the two values of the effective absorption strength *γ*: 19.4 ± 3.8 (red open circles) and 48.4 ± 4.5 (red full circles) are shown for the microwave networks with *T* invariance violation. They are obtained by averaging over 250 and 251 realizations of the networks containing 1 dB and 2 dB attenuators, respectively. The total “optical” length of the networks including edges, phase shifters, joints, attenuators and circulators, was varied, depending on the network configuration, from 777 cm to 912 cm.

Figure 3 presents also the corresponding theoretical distributions *P*(*R*) (green and blue solid lines) calculated from the Eq. (5) for the parameter *γ* = 19.4 and *γ* = 48.4, respectively. An overall very good agreement of the experimental distributions *P*(*R*) with the theoretical ones confirms that the procedure leading to the determination of the strength parameter *γ* works very well for systems with violated *T* invariance.

In the inset in Fig. 3 we show the distributions of the experimental reflection coefficient *P*(*R*) in semi-log scale. The straight lines *ln*(*P*(*R*)) = *aR* + *b* fitted to the experimental results yielded the slopes *a* = −19.6 ± 0.2 and *a* = −48.9 ± 0.5 for the networks with 1 dB and 2 dB attenuators, respectively. These results clearly demonstrate that we deal with large absorption regime described with good approximation by the exponential distribution (7) with the exponent *α* = −*a*.

In Table 1 the enhancement factor *W* of the scattering matrix $\hat{S}$ of the microwave networks measured for two experimental values of the parameter *γ* is compared to the theoretical prediction *W*_*th*_. The enhancement factor *W*_*th*_ was calculated using the formula (4) by applying the formulas (19) in ref.^56^ for the variances of the scattering matrix elements *S*_*ij*_. In the calculations we used experimentally measured transmission coefficients^56^ *T*_1_ = 0.58 ± 0.08 and *T*_2_ = 0.43 ± 0.05. The internal absorption strength *γ*_*abs*_ was calculated from the formula *γ*_*abs*_ = *γ* − *T*_1_ − *T*_2_. The comparison of the experimental and theoretical results clearly shows that we deal with the system with broken *T* invariance.

**Table 1:** The experimental enhancement factor *W* of the microwave graphs with violated time reversal symmetry compared to the theoretical one *W*_*th*_ for two experimental values of the effective parameter *γ*.

*γ*	*W*	*W*_*th*_
19.4 ± 3.8	1.01 ± 0.07	1.04
48.4 ± 4.5	1.04 ± 0.10	1.03

**Figure 4:**
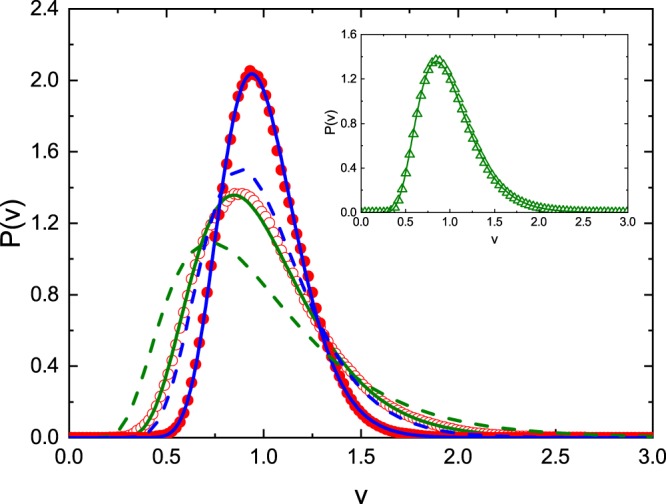
Experimentally evaluated distributions of the imaginary part of the diagonal elements of the Wigner’s matrix *P*(*ʋ*) for the microwave graphs with violated *T* invariance for two values of the mean absorption parameter *γ* = 19.4 (red open circles) and *γ* = 48.4 (red full circles). The theoretical distributions *P*(*ʋ*) evaluated from the Eq. (8) are marked by green (*γ* = 19.4) and blue (*γ* = 48.4) solid lines, respectively. The theoretical distribution *P*(*ʋ*) for the GOE systems (with *T* invariance) for the same values of the parameter *γ* = 19.4 and *γ* = 48.4 are shown with green and blue dashed lines, respectively. The inset shows the comparison of the theoretical distribution *P*(*ʋ*) calculated for the parameter *γ* = 19.4 from the exact formula (8) (green solid line) with the approximated one evaluated from the formula (13) (green triangles).

Figure 4 shows the experimentally evaluated distributions of the imaginary part of the diagonal elements of the Wigner’s reaction matrix *P*(*ʋ*) for the microwave graphs with *T* invariance violation. The results are attained for the two values of the parameter *γ* = 19.4 (red open circles) and *γ* = 48.4 (red full circles), respectively. The experimental results presented in Fig. 4 are generally in good agreement with the theoretical ones denoted by green and blue solid lines, respectively. However, the experimental distribution for *γ* = 19.4 in the vicinity of the maximum is slightly shifted towards higher values of the parameter *v*. In Fig. 4 we also show the theoretical distribution *P*(*ʋ*) for the GOE systems (with *T* invariance) for the same values of the parameter *γ* = 19.4 (green dashed line) and *γ* = 48.4 (blue dashed line), respectively. We clearly see that the distributions *P*(*ʋ*) for the systems with violated *T* invariance are significantly more peaked than the ones with *T* invariance. They are also shifted towards higher values of the parameter *v*. In the inset in Fig. 4 we show the comparison of the theoretical distribution *P*(*ʋ*) calculated for the parameter *γ* = 19.4 using the exact formula (8) (green solid line) with the approximated one evaluated from the formula (13) (green triangles). Again we see very good agreement between the approximated and exact results confirming that we really work in the regime of large absorption.

**Figure 5:**
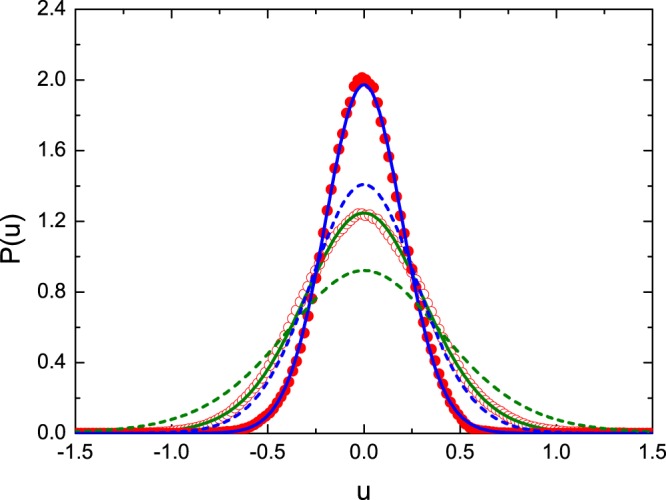
Experimentally evaluated distributions of the real part of the diagonal elements of the Wigner’s matrix *P*(*u*) for the microwave graphs with violated *T* invariance for two values of the mean absorption parameter *γ* = 19.4 (red open circles) and *γ* = 48.4 (red full circles). The experimental results are compared to the theoretical ones evaluated from the Eq. (12): green (*γ* = 19.4) and blue (*γ* = 48.4) solid lines, respectively. The theoretical distributions *P*(*u*) for the systems with *T* invariance for the same values of the parameter *γ* = 19.4 and *γ* = 48.4 are shown with green and blue dashed lines, respectively.

In Fig. 5 the distributions of the real part of the diagonal elements of the Wigner’s matrix *P*(*u*) are shown for the microwave graphs for the two values of the parameter *γ* = 19.4 (red open circles) and *γ* = 48.4 (red full circles), respectively. The experimental distributions are compared to the theoretical ones which are marked by green and blue solid lines, respectively, evaluated from the Eq. (8). In general, good agreement between the experimental and theoretical results is observed. However, one may indicate that in the vicinity of the maximum (−0.1 < *u* < 0.1) the experimental distribution *P*(*u*) for *γ* = 48.4 lies slightly above the theoretical one. Such a behavior of the experimental distribution *P*(*u*) suggests the excess of small values of the imaginary part of the normalized impedance |*Imz*_*ii*_|, whose origin is not known. For comparison, in Fig. 5 we show the theoretical distribution *P*(*u*) for the GOE systems for the same values of the parameter *γ* = 19.4 (green dashed line) and *γ* = 48.4 (blue dashed line), respectively. Similarly to the situation for the distributions *P*(*ʋ*) the distributions *P*(*u*) for the systems with violated *T* invariance are significantly more peaked than the ones with *T* invariance. One should point out that also in this case the agreement between the exact formula (9) and the approximated one (11) was excellent. We do not show the inset with this comparison because the former results obtained for *P*(*R*) and *P*(*ʋ*) distributions have already very firmly proved that we deal with the regime of large absorption.

## Conclusions

We present the first experimental investigation of the distributions *P*(*ʋ*) and *P*(*u*) of the imaginary and the real parts of the diagonal elements of the Wigner’s $\hat{K}$ matrix for irregular microwave graphs with broken time-reversal invariance in the case of large absorption. We showed that the experimentally evaluated distributions *P*(*ʋ*) and *P*(*u*) are in good agreement with the theoretical ones. In this study the effective absorption strength *γ* was evaluated using the distribution of the reflection coefficient *P*(*R*). However, *a posteriori*, we clearly see that the distributions *P*(*ʋ*) and *P*(*u*) can also be used for the same purpose. Their advantage over the reflection coefficient *P*(*R*) stems from the fact that in the case of the diagonal elements of the Wigner’s reaction $\hat{K}$ matrix we deal with a self-consistent method which simultaneously uses both distributions *P*(*ʋ*) and *P*(*u*). Therefore, our results suggest that the distributions *P*(*ʋ*) and *P*(*u*) of the imaginary and the real parts of the diagonal elements of the Wigner’s reaction $\hat{K}$ matrix together with the enhancement factor *W* can be used as a very powerful tool for identification of systems with violated *T* symmetry and evaluation of their absorption strength *γ*. This is especially important in the limit of large absorption where the other measures connected with the short- and long-range spectral correlation functions cannot be applied.
